# Supplementary material for: A fault-intrusion-tolerant system and deadline-aware algorithm for scheduling scientific workflow in the cloud
Source: PeerJ Comput Sci. 2021 Nov 2;7:e747. doi: 10.7717/peerj-cs.747 (PMC8576554; doi:10.7717/peerj-cs.747)
Supplement: Supplemental Information 6 [file peerj-cs-07-747-s006.zip › Myproject1.1/docs/org/workflowsim/failure/FailureRecord.html]

FailureRecord


JavaScript is disabled on your browser.


- Overview
- Package
- Class
- Use
- Tree
- Deprecated
- Index
- Help

- Prev Class
- Next Class

- Frames
- No Frames

- All Classes

- Summary:
- Nested |
- Field |
- Constr |
- Method

- Detail:
- Field |
- Constr |
- Method


org.workflowsim.failure

## Class FailureRecord

- java.lang.Object
- - org.workflowsim.failure.FailureRecord

- ---

    

  ```
  public class FailureRecord
  extends java.lang.Object
  ```

  Failure Record is a record with all information of a failure

  Since:
  :   WorkflowSim Toolkit 1.0

- - ### Field Summary

    Fields

    | Modifier and Type | Field and Description |
    | --- | --- |
    | `int` | `allTaskNum` all the tasks (failed or not). |
    | `double` | `delayLength` delay length. |
    | `int` | `depth` the depth (also used as type in some cases) of a failure. |
    | `int` | `failedTasksNum` number of failed tasks. |
    | `int` | `jobId` the job id. |
    | `double` | `length` Length |
    | `int` | `vmId` the location. |
    | `int` | `workflowId` the workflow id (user id in this version). |
  - ### Constructor Summary

    Constructors

    Constructor and Description

    `FailureRecord(double length,
    int tasks,
    int depth,
    int all,
    int vm,
    int job,
    int workflow)`

    Initialize a Failure Record
  - ### Method Summary

    - ### Methods inherited from class java.lang.Object

      `clone, equals, finalize, getClass, hashCode, notify, notifyAll, toString, wait, wait, wait`

- - ### Field Detail


    - #### length

      ```
      public double length
      ```

      Length


    - #### failedTasksNum

      ```
      public int failedTasksNum
      ```

      number of failed tasks.


    - #### depth

      ```
      public int depth
      ```

      the depth (also used as type in some cases) of a failure.


    - #### allTaskNum

      ```
      public int allTaskNum
      ```

      all the tasks (failed or not).


    - #### vmId

      ```
      public int vmId
      ```

      the location.


    - #### jobId

      ```
      public int jobId
      ```

      the job id.


    - #### workflowId

      ```
      public int workflowId
      ```

      the workflow id (user id in this version).


    - #### delayLength

      ```
      public double delayLength
      ```

      delay length.
  - ### Constructor Detail


    - #### FailureRecord

      ```
      public FailureRecord(double length,
                   int tasks,
                   int depth,
                   int all,
                   int vm,
                   int job,
                   int workflow)
      ```

      Initialize a Failure Record

      Parameters:
      :   `length,` - length of this task
      :   `tasks,` - number of failed tasks
      :   `depth,` - depth of the failed tasks
      :   `all,` - all the tasks
      :   `vm,` - vm id where the failure generates
      :   `job,` - job id where the failure generates
      :   `workflow` - , workflow id where the failure generates


- Overview
- Package
- Class
- Use
- Tree
- Deprecated
- Index
- Help

- Prev Class
- Next Class

- Frames
- No Frames

- All Classes

- Summary:
- Nested |
- Field |
- Constr |
- Method

- Detail:
- Field |
- Constr |
- Method
